# Uncommon Manifestation: Thrombotic Thrombocytopenic Purpura Presenting Solely as Petechial Rash

**DOI:** 10.7759/cureus.57994

**Published:** 2024-04-10

**Authors:** Faryal Altaf, Shitij Shrivastava, Rachana Allena, Jai Kumar, Misbahuddin Khaja

**Affiliations:** 1 Internal Medicine, Icahn School of Medicine at Mount Sinai/Bronx Care Health System, New York, USA; 2 Medicine, California Institute of Behavioral Neurosciences & Psychology, Fairfield, USA; 3 Internal Medicine/Pulmonary Critical Care, Icahn School of Medicine at Mount Sinai/Bronx Care Health System, New York, USA

**Keywords:** thrombocytopenia, rituximab, immunosuppressive therapy, plasmapheresis, microvascular occlusion, platelet aggregation, microangiopathic hemolytic anemia, adamts13 deficiency, petechial rash, thrombotic thrombocytopenic purpura (ttp)

## Abstract

Thrombotic thrombocytopenic purpura (TTP) is a rare, life-threatening disorder typically presenting with a classic pentad of symptoms: thrombocytopenia, microangiopathic hemolytic anemia, neurological abnormalities, renal dysfunction, and fever. This report explores an unusual presentation of TTP in a 47-year-old female with a medical history of hypertension, hyperlipidemia, and chronic TTP, who exhibited only petechial rashes, generalized weakness, and headache. Notably, the petechial rash, a less common manifestation of TTP, became a pivotal clue for the diagnosis, underscoring the necessity for vigilance even when classic symptoms are absent. This case reinforces the imperative of a high suspicion index for TTP, especially in patients with thrombocytopenia and hemolytic anemia, irrespective of other traditional signs. Plasmapheresis remains the treatment cornerstone, removing autoantibodies and replenishing ADAMTS13, as evidenced by the patient's initial response. The administration of rituximab, targeting B cells to mitigate autoantibody production against ADAMTS13, featured prominently in her management, aligning with its recognized role in refractory or relapsing TTP cases. Despite an encouraging response to rituximab, a subsequent decline in platelet count indicated the unpredictable nature of TTP and the necessity for multi-pronged therapeutic strategies. The patient's medical background and persistently low ADAMTS13 levels hinted at a chronic relapsing trajectory associated with increased morbidity and mortality. This necessitates ongoing vigilance and treatment flexibility. Highlighting this atypical TTP presentation, the report calls for immediate, robust intervention, serving as a critical reminder of the heterogeneity of TTP manifestations and the complexities in its management, thereby contributing to broader clinical awareness and improved patient prognoses.

## Introduction

Thrombotic thrombocytopenic purpura (TTP) is a rare hematologic emergency defined by a characteristic pentad: thrombocytopenia, microangiopathic hemolytic anemia, neurological complications, renal impairment, and fever [1]. The pathophysiology hinges on a pronounced deficiency of ADAMTS13, a von Willebrand factor-cleaving protease. This deficiency triggers the accumulation of abnormally large von Willebrand factor multimers, which promote platelet adhesion and aggregation [2]. It results in microthrombi forming within small blood vessels, leading to ischemic organ damage and clinical manifestations [3]. One of the hallmark signs of TTP is the appearance of petechial rashes resulting from thrombocytopenia and microvascular occlusions associated with the disease. While it is not considered a typical manifestation of the disease, it is seen in many cases [4]. Such atypical manifestations necessitate heightened clinical vigilance to ensure prompt and accurate diagnosis, which is critical for initiating life-saving interventions [5]. The mainstay of treatment for TTP is plasma exchange, which has significantly improved the prognosis of the disease by removing circulating autoantibodies and replenishing deficient ADAMTS13 [6]. Adjuvant immunosuppression, with agents such as corticosteroids and rituximab, has also proved to be an effective strategy in TTP management, reducing autoantibodies [7]. However, the role of these agents in atypical presentations of TTP, such as those with petechial rash, needs to be better established [8].

In this case report, we detail the atypical presentation of a 47-year-old female with a previous episode of TTP, presented with a singular finding of petechial rashes diagnosed with TTP, necessitating urgent medical intervention. This case highlights the critical nature of TTP, the importance of recognizing atypical presentations, and the need for immediate and decisive action in managing this complex hematologic disorder.

## Case presentation

**Figure 1:**
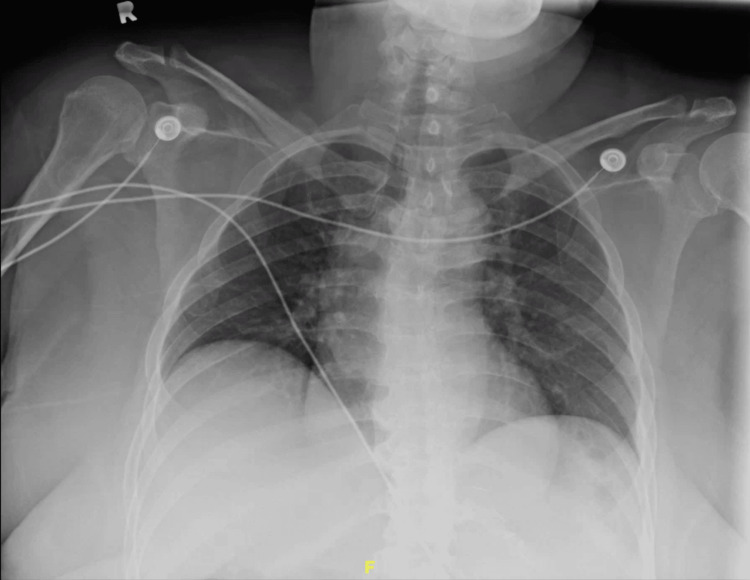
Xray of the chest showing normal anatomy

**Figure 2:**
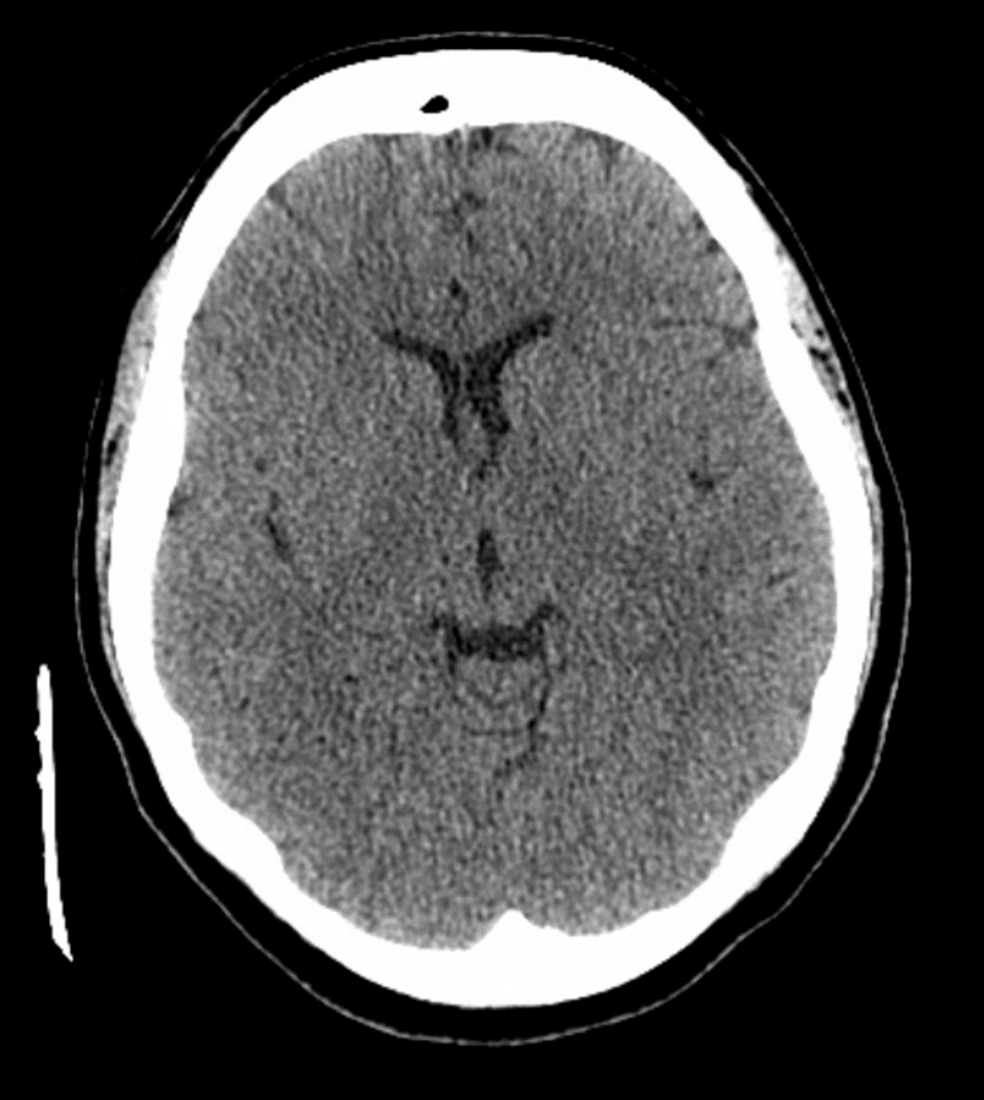
CT of the head showing normal architecture.

A 47-year-old female with a notable medical history of hypertension compliant with medication, hyperlipidemia, and TTP came to the emergency department, reporting a two-day history of petechial rash without any fever. Initially manifesting on her fingers and toes, the rash spread across her body. She also reported experiencing generalized weakness and a headache. Of particular concern was her report of heavy menstruation, necessitating the use of 14 sanitary pads per day, a significant increase from her usual five pads per day. She denied experiencing any gum bleeding, joint swelling or pain, hematemesis, hemoptysis, melena, or hematuria. Physical examination revealed no notable findings apart from the petechial rash. X-rays of the chest and CT head were done within normal limits, as shown in Figures 1, 2.

Laboratory investigations highlighted severe thrombocytopenia, a platelet count of 16 k/μL, and anemia with hemoglobin 9.4. Initial laboratory investigations are mentioned in Table 1. The patient had a previous admission for similar symptoms three years prior, during which she was diagnosed with TTP, treated with plasmapheresis, and subsequently discharged following an improvement in her thrombocytopenia. Her ADAMTS13 activity levels during the acute episode were notably low at three but increased to 56 several months post plasmapheresis.

**Table 1:** Summary of laboratory investigations. BUN: blood urea nitrogen; PT: prothrombin time; INR: international normalised ratio; APTT: activated partial thromboplastin time

Laboratory Test	Test Values	Reference Values
Serum Creatinine	0.8	0.50-1.10 mg/dl
BUN	19	8.0-26.0 mg/dl
Hemoglobin	9.4	12-16g/dl
White Blood Cell Count	8.3	4.8-10.8k/ul
Platelet Count	16	150-400 k/ul
PT	12	9.9-13.3 seconds
INR	1.03	0.85-1.14
Total Bilirubin	1.7	0.2-1.2 mg/dL
APTT	35.6	25.1-36.5 seconds
Total Serum Calcium	8.4	8.5-10.5 mg/dL
Direct Bilirubin	0.4	0.0-0.3 mg/dL
Lactate Dehydrogenase	1246	100-190 unit/L
Haptoglobin	<10	30-200 mg/dL
ADAMTS13 Activity	<0.03	68-163 IU/mL
ADAMTS13 Inhibitor	2.9	<0.4 BEU
D-Dimer	1797	0-230 ng/mL
Fibrinogen	322	185–450 mg/dL

**Figure 3:**
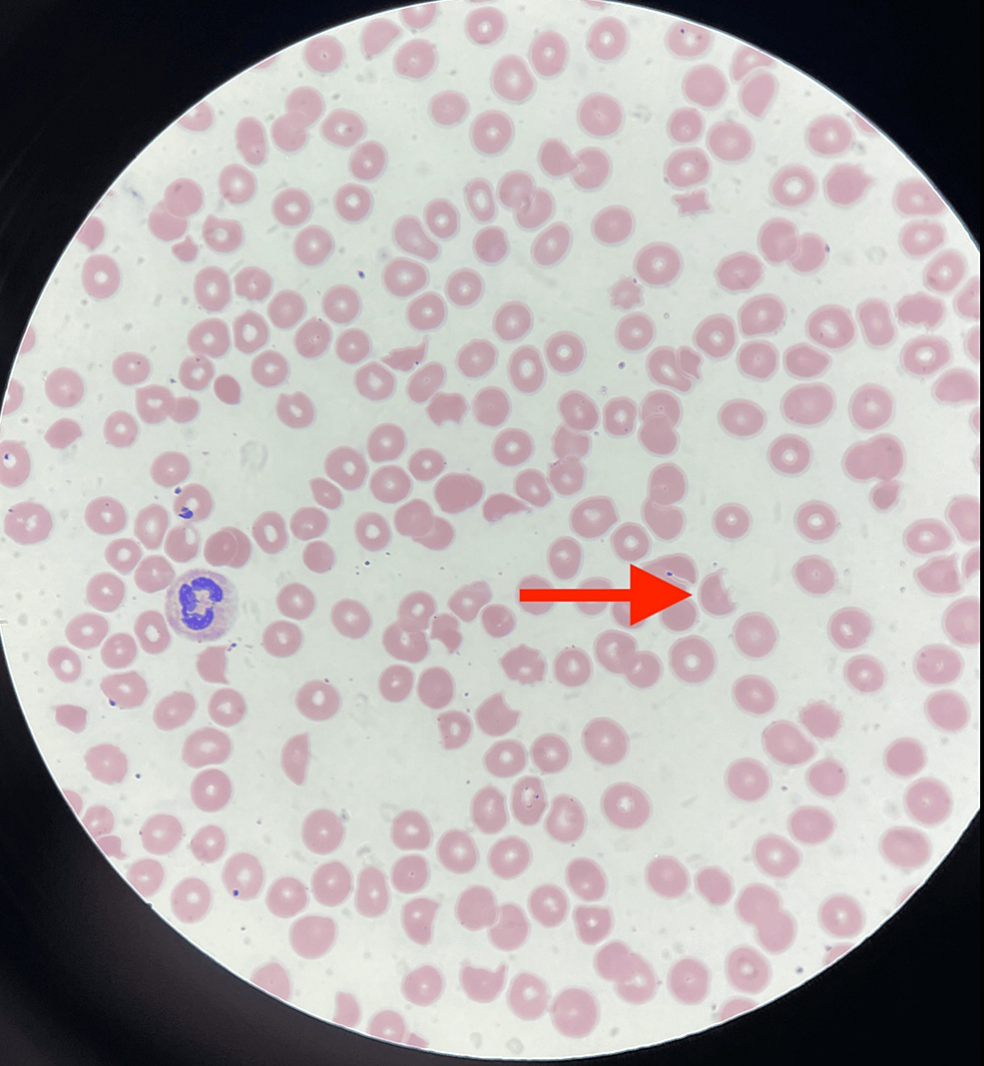
Peripheral smear showing schistocytes (red arrow).

During her hospitalization, further laboratory workup confirmed hemolytic anemia and severe thrombocytopenia, prompting consultation with the hematology department. She was initiated on intravenous steroids, 1 mg/kg per day. The absence of heparin or heparin products in her recent medical history excluded heparin-induced thrombocytopenia (HITT) as a differential diagnosis. The presence of schistocytes in a peripheral blood smear led to a reaffirmed diagnosis of TTP, as shown in Figure 3. On the second day of her admission, the patient was transferred to the intensive care unit (ICU) for the commencement of plasmapheresis while continuing intravenous steroids. Plasmapheresis was continued for four days. There was negligible improvement in her thrombocytopenia despite completing four days of plasmapheresis, leading to the discontinuation of plasmapheresis. A strategic decision was made against platelet transfusion due to the potential worsening of TTP symptoms and the risk of seizures. 

**Figure 4:**
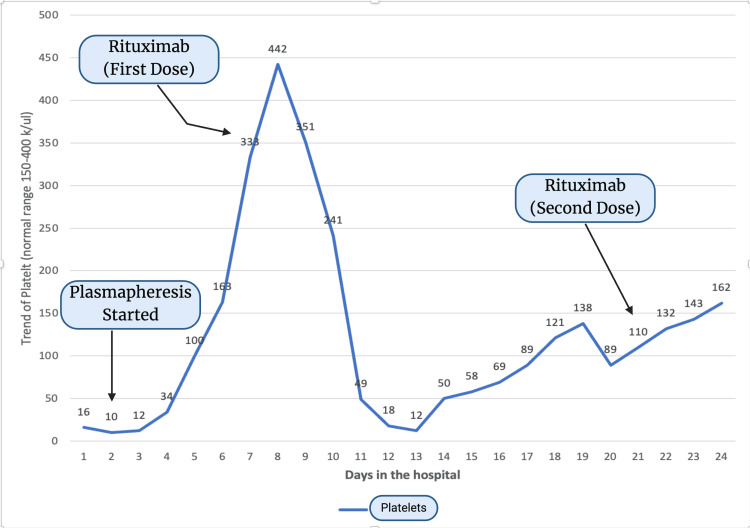
The trend of platelets during the hospital course x-axis shows days in the hospital; y-axis shows platelet values

Subsequent ADAMTS13 activity level testing revealed critically low levels (<10), confirming the TTP diagnosis. The treatment strategy was thus adjusted to include immunosuppressants. The patient received a dose of rituximab 375 mg/m^2^ on the seventh day of admission. This resulted in an initial improvement in thrombocytopenia that significantly declined within three days, necessitating a second dose of rituximab. She continued intravenous steroids for 10 days before transitioning to oral steroids, namely prednisone 40 mg. After administering the second rituximab dose, a marked improvement in her platelet count was observed. The gynecology department evaluated her for menorrhagia but did not recommend any specific intervention. She was discharged on oral steroids with instructions for outpatient follow-up in the hematology clinic. Eight months post discharge, her ADAMTS13 activity level was re-evaluated, showing a return to normal at 64. The trend of platelets during the hospital course is shown in Figure 4.

## Discussion

This case report underscores the importance of recognizing the atypical presentations of TTP, mainly when a patient presents with petechial rashes [9]. While not classically associated with TTP, this symptom should elicit consideration of the condition, especially when accompanied by thrombocytopenia and microangiopathic hemolytic anemia [10]. Petechial rashes in TTP can be attributed to thrombocytopenia and microvascular thrombosis, reducing blood flow to the skin. This highlights the importance of maintaining a high index of suspicion for TTP in patients presenting with thrombocytopenia and microangiopathic hemolytic anemia, even without the classic clinical features [11,12].

Plasmapheresis is the cornerstone of TTP treatment, aiming to remove autoantibodies and replenish ADAMTS13, an enzyme crucial for cleaving von Willebrand factor and preventing platelet aggregation [13]. The initial improvement in the patient's condition following plasmapheresis aligns with existing literature supporting the efficacy of this treatment in TTP, which has been shown to improve survival significantly [14]. Notably, the use of rituximab emerged as a significant element in the treatment regimen for the present case, reflecting its growing recognition as an effective therapy, particularly in TTP cases that are refractory or have a propensity for relapse [15].

The role of rituximab in this case is noteworthy, as it has been increasingly recognized as a valuable therapeutic option in TTP management, particularly in refractory or relapsing cases [16,17]. However, the subsequent decline in platelet count despite Rituximab therapy underscores the need for a multifaceted treatment approach and the potential for disease recurrence, even in patients responding well to initial treatment. Despite the initial lack of response to plasmapheresis, the patient's improvement following rituximab administration highlights the potential benefits of this therapeutic agent in managing TTP [18]. This patient’s previous history of TTP and plasmapheresis, along with the low ADAMTS13 levels, suggest a chronic or relapsing form of TTP, which has been associated with higher morbidity and mortality [19].

This case underscores the importance of considering TTP in patients presenting with atypical symptoms such as petechial rashes and menorrhagia. This case further highlights the potential for a chronic or relapsing trajectory in TTP, evidenced by persistently low ADAMTS13 activity, which carries a higher risk of morbidity and mortality. Early recognition and prompt initiation of appropriate therapies, including plasmapheresis, steroids, and rituximab, are vital for improving outcomes in this potentially fatal condition [20]. Continuous monitoring and adjusting treatment plans are indispensable for navigating TTP's complexities and enhancing the quality of life for those affected by this severe condition.

## Conclusions

The current case of TTP presenting solely as a petechial rash emphasizes the disease's diagnostic and therapeutic challenges. Despite an initial improvement with plasmapheresis and rituximab, the patient's subsequent platelet count decline underscores TTP's unpredictable course and the need for an adaptable management strategy. The patient's chronic TTP, indicated by low ADAMTS13 levels, called for ongoing vigilance and flexibility in treatment. This report highlights the importance of considering TTP in patients with atypical symptoms such as isolated petechial rashes. Early and aggressive treatment interventions are essential for improving outcomes. Continuous monitoring and readiness to revise treatment approaches are vital in managing TTP, which can deviate from its classic presentation. Ultimately, this case serves as a pivotal reminder of the variability of TTP manifestations and underscores the imperative for clinical awareness to optimize patient care and prognosis.
